# Multilayer Semantic Features Adaptive Distillation for Object Detectors

**DOI:** 10.3390/s23177613

**Published:** 2023-09-02

**Authors:** Zhenchang Zhang, Jinqiang Liu, Yuping Chen, Wang Mei, Fuzhong Huang, Lei Chen

**Affiliations:** 1 Key Laboratory of Smart Agriculture and Forestry, College of Computer and Information Sciences, Fujian Agriculture and Forestry University, Fuzhou 350002, China; 2 College of Mechanical and Electrical Engineering, Fujian Agriculture and Forestry University, Fuzhou 350002, China; 3 Key Laboratory of Marine Biotechnology of Fujian Province, Institute of Oceanology, Fujian Agriculture and Forestry University, Fuzhou 350002, China

**Keywords:** multilayer semantic feature, knowledge distillation, object detection, adaptive distillation

## Abstract

Knowledge distillation (KD) is a well-established technique for compressing neural networks and has gained increasing attention in object detection tasks. However, typical object detection distillation methods use fixed-level semantic features for distillation, which might not be best for all training stages and samples. In this paper, a multilayer semantic feature adaptive distillation (MSFAD) method is proposed that uses a routing network composed of a teacher and a student detector, along with an agent network for decision making. Specifically, the inputs to the proxy network consist of the features output by the neck structures of the teacher and student detectors, and the output is a decision on which features to choose for distillation. The MSFAD method improves the distillation training process by enabling the student detector to automatically select valuable semantic-level features from the teacher detector. Experimental results demonstrated that the proposed method increased the mAP_50_ of YOLOv5s by 3.4% and the mAP_50–90_ by 3.3%. Additionally, YOLOv5n with only 1.9 M parameters achieved detection performance comparable to that of YOLOv5s.

## 1. Introduction

In recent years, deep neural networks have been widely adopted in various fields [1,2,3,4,5], with increasingly complex model structures designed to achieve higher performance. However, these models require substantial computing resources and have very low inference speeds. Knowledge distillation (KD) [6] has been proposed to solve those problems. KD is a highly effective neural network compression method that transfers the dark knowledge contained in a bulky teacher model to a compact student model, enabling the latter to achieve advanced performance. Relative to other compression methods [7,8,9,10], KD minimizes the loss in performance caused by compression and requires no special hardware or software support.

**Figure 1:**
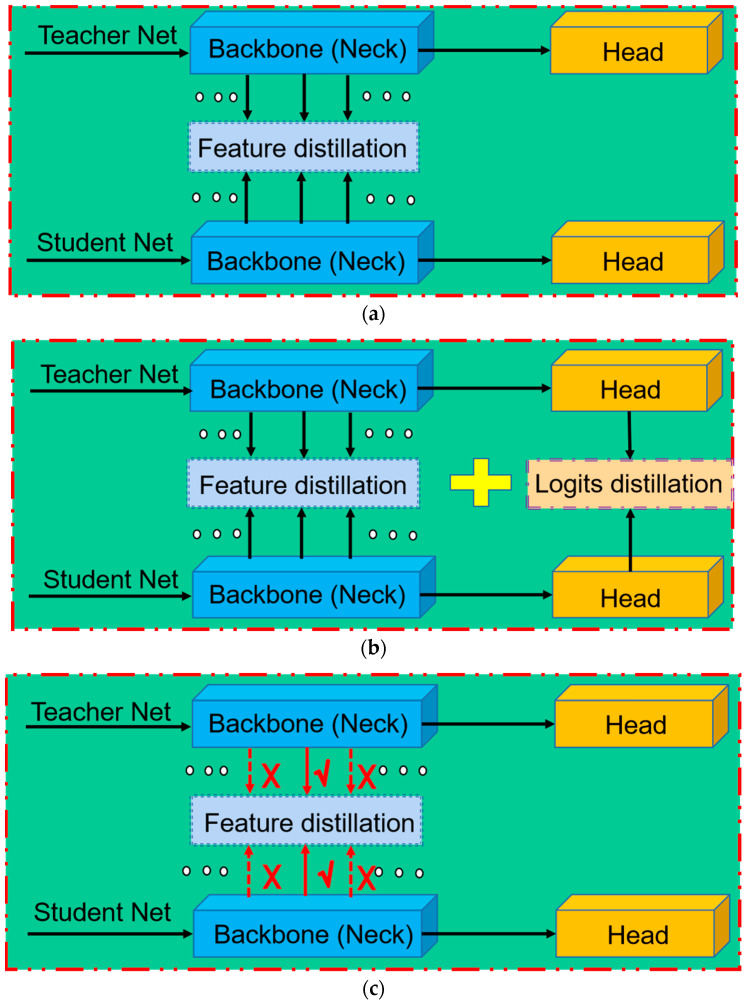
Overview of existing object detection distillation methods. (**a**) Conventional approach, where the distillation framework focuses solely on fixed semantic-level features of detectors. (**b**) An extension of (**a**) that incorporates logit distillation while keeping the semantic feature level fixed. (**c**) Proposed multilayer semantic feature adaptive distillation method (MSFAD), which uses adaptive semantic-level features for distillation. The red dotted lines indicate that features at that semantic level are currently not used for distillation in the current training stage and samples.

After substantial progress in recent years, KD methods for image classification tasks have matured [11,12,13,14,15]. However, object detection tasks require consideration of both classification and localization, and there is an imbalance between foreground and background issues [16]. Hence, important challenges persist in using KD for object detection tasks. Therefore, several recent studies have focused on adapting KD methods for object detection tasks [17,18,19,20,21]. As shown in Figure 1a,b, those studies can be divided into two primary categories:

(1) Distilling only the specific semantic-level features of the detector [18,19,21] (Figure 1a). For example, [18] used the region proposal network structure of the student detector to select positive regions from the fixed-level features, and [21] distilled the foreground and background of the intermediate layer features separately.
(2) Distilling the specific semantic-level features of the detector and the logit output by the detector head [17,20] (Figure 1b). For example, in [20], semantic features were distilled independently from the backbone network, classification head, and regression head.

Those methods all used a fixed semantic level of features for distillation, which did not change during training.

However, ref. [22] noted that when a classifier is distilled, the distillation point’s location should be adjusted in accordance with different training stages and samples. Therefore, ref. [22] proposed a spot-adaptive distillation method, which has been shown to improve the performance of distillation methods for classification tasks.

Inspired by [22], in the present study, where to distill was also considered when distilling detectors, which has been disregarded by most current object detection distillation methods. This paper proposes a multilayer semantic feature adaptive distillation (MSFAD) method for object detectors to address the problems above. As shown in Figure 1c, the proposed method diverges from the object detection distillation approaches shown in Figure 1a,b. It empowers the student detector to autonomously discern and incorporate valuable semantic-level features from the teacher detector, depending on the training stage and samples. Specifically, the MSFAD approach uses a routing network for teacher and student detectors and an agent network for decision making. The proxy network takes the features output by the neck structures of the teacher and student detectors as input and determines whether to use the current semantic-level features for distillation.

There are two differences between our approach and that of [22]. First, all features directly fed into the detector head are adopted as input to the proxy network rather than only the last layer features of the teacher and student models. This decision was made since mainstream detectors [3,4,23,24,25,26,27,28] usually use multiscale feature fusion [29], which inputs features of various scales into the detection head to detect objects of varying sizes. Thus, the input features of the detector head often come from multiple semantic levels. In this study, it was found that using all features input into the detector head in the proxy network resulted in better decisions for final detection. Second, to ensure method generality, only the semantic features of the middle levels were distilled since different detectors have varying head structures. In summary, the contributions of this paper are:

(1) A novel MSFAD method is proposed for object detectors that addresses the problem in current object detection distillation methods of the mismatch between the semantic level of distilled features and the training stage and samples.
(2) The selection of various semantic-level features for distillation at different training stages is described, and the important effect of semantic-level selection during distillation training is highlighted.
(3) The experiments described show that the MSFAD method improved the mAP_50_ and mAP_50–90_ of YOLOv5s by 3.4% and 3.3%, respectively. Moreover, it is demonstrated that MSFAD achieved detection performance similar to YOLOv5s for YOLOv5n with only 1.9 M parameters. Relative to the latest YOLOv7-tiny of the same magnitude, the YOLOv5s model distilled by our method achieved higher mAP_50_ and mAP_50–90_ by 2.2% and 1.9%, respectively.

The paper is organized as follows: Section 2 reviews related work on object detection algorithms and KD algorithms. Section 3 presents the proposed semantic-level adaptive distillation algorithm. Section 4 details the experimental process and presents experimental results. Finally, Section 5 provides a conclusion.

## 2. Related Work

### 2.1. Object Detection

Object detection is a fundamental task in computer vision widely applied in various scenarios [30,31,32,33,34]. Currently, deep learning methods are the mainstream approach for object detection. Object detection methods can be categorized into three groups: (1) Two-stage object detection [3,4,28], (2) single-stage object detection based on anchor boxes [23,24,25,26,27,35], and (3) single-stage object detection that is anchor free [36,37], following various detection principles.

Two-stage object detection algorithms, such as the region-based convolutional neural network (R-CNN) family [3,4,28], first extract object areas and then classify the extracted object areas. However, the main disadvantage of that method is its slow inference speed, which limits its practical application.

In contrast, the “you only look once” (YOLO) single-stage detection algorithm, proposed by Redmon et al. [23] in 2016, directly outputs the position information and category of the prediction frame at the output layer, which greatly improves the model’s inference speed. Various improvements on the basis of YOLOv1 have been made [24,25,26,27,35]. Consequently, the YOLO family has become the preferred algorithm for various detection tasks. However, most object detection distillation methods adopt Faster-RCNN as the benchmark model, which is not proven to be suitable for YOLO. Therefore, this study uses YOLOv5 as the benchmark model for object detection distillation methods.

Two researchers have used anchor-free methods to complete target detection tasks. Duan et al. [36] modeled object detection as a center-point detection problem, combining prediction of center points and bounding boxes to achieve efficient and accurate object detection. Tian et al. [37] achieved the efficiency and accuracy of one-stage object detection by the use of innovative methods, such as a complete convolutional structure, center-width height representation, and adaptive receptive fields.

### 2.2. Knowledge Distillation

Knowledge distillation is a compression technique that does not modify the network architecture. The fundamental concept behind the approach is to transfer the “dark” knowledge from a larger teacher model to a smaller student model to attain similar performance. Hinton et al. [6] first introduced minimizing the KL divergence between teacher and student probability outputs to improve student performance. Later work [11] indicated that using both semantic features from middle layers and logits for distillation could lead to greater improvements. Later studies [12,13,14,15] markedly improved the student classifier’s performance. Today, KD has emerged as a well-established compression method for classification tasks.

However, classification and localization problems must be considered for object detection tasks since they often lead to a marked imbalance between foreground and background objects [16]. Therefore, directly applying classification distillation methods to detection tasks is not ideal. Several studies [17,18,19,20,21,38] have recently attempted to apply KD to object detection tasks, improving student detectors’ performance. For instance, Chen et al. [17] used hint learning [11] to distill the semantic features of the intermediate layers of a detector by designing distillation weights to suppress the background. Li et al. [18] used the region proposal network structure of the student detector to extract semantic features from the middle layer and then distilled the extracted positive feedback regions. Similarly, Sun et al. [20] distilled semantic features from the backbone network, classification head, and regression head separately, and Dai et al. [38] introduced a relation-based distillation method to simultaneously distill semantic features and detection head logits of the middle layer of the teacher detector. Additionally, attention mechanisms have been used in various fields [39,40,41], and Yang et al. [21] incorporated the attention mechanism to enable the student detector to focus on useful local pixels while introducing global distillation to compensate for the lack of pixel relationships.

In conclusion, the object detection distillation methods mentioned above can be categorized into two types: (1) Those that distill intermediate layer features based solely on semantics [18,19,21], and (2) those that distill intermediate layer features and detection head logits simultaneously [17,20,38]. Once the semantic level of the features used for distillation is established in those methods, it remains unchanged throughout the distillation training process. Those approaches prioritize what to distill over where to distill.

## 3. Method

**Figure 2:**
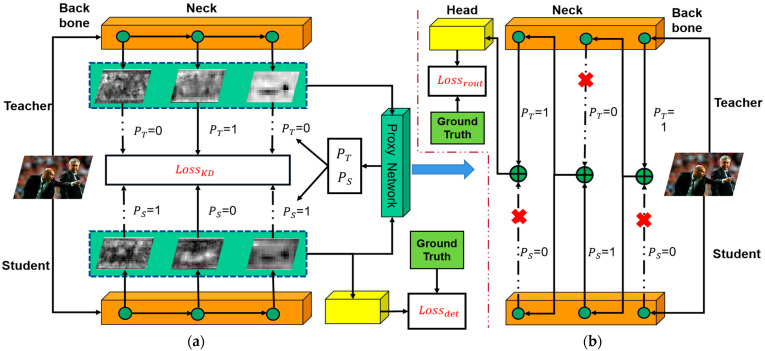
Overall framework of proposed multilayer semantic feature adaptive distillation. The training process of MSFAD comprises two forward processes. (**a**) Shows the distillation feedforward process. It computes the detection loss of the student detector ${\mathrm{Loss}}_{\det}$ and the distillation loss of the feature ${\mathrm{Loss}}_{\mathrm{KD}}$ and acquires the decision of the proxy network P = (P_T_, P_S_). (**b**) Shows the routing feedforward process, which establishes the feedforward path by leveraging the decision made by the proxy network. It then computes the routing loss ${\mathrm{Loss}}_{\mathrm{rout}}$ using the output from the teacher detector’s head and the ground truth. The dashed arrow indicates that the semantic features of the layer are not selected.

The proposed MSFAD method (Figure 2), which involves two forward propagation processes, is introduced in this section. The first process (Figure 2a) is the distillation feedforward, focused primarily on calculating the detection and distillation losses of the student detector. The distillation loss calculation is constrained by the output of the proxy network. $P_{T}$ = 1 indicates distillation using features from the current semantic level, whereas $P_{T}$ = 0 implies no distillation. Due to the interdependencies between the neck structure output and the proxy network’s decisions, training of both networks simultaneously can lead to training failure. To address that problem, the second process, routing feedforward, is used (Figure 2b). The routing path is determined by the proxy network’s decision during the initial feedforward. If $P_{T}$ = 1, the teacher detector’s features are used as input for the next routing network layer. If $P_{S}$ = 1, the student detector’s features are used instead. The input features of the subsequent layer are aligned in channel dimensions through a 1 × 1 convolution operation. Since the teacher detector has already completed training before distillation training, the output of the teacher detector serves as the final output of the routing network. The routing loss is calculated by comparing that output with the ground truth value.

### 3.1. Distillation Feedforward Process

**Figure 3:**
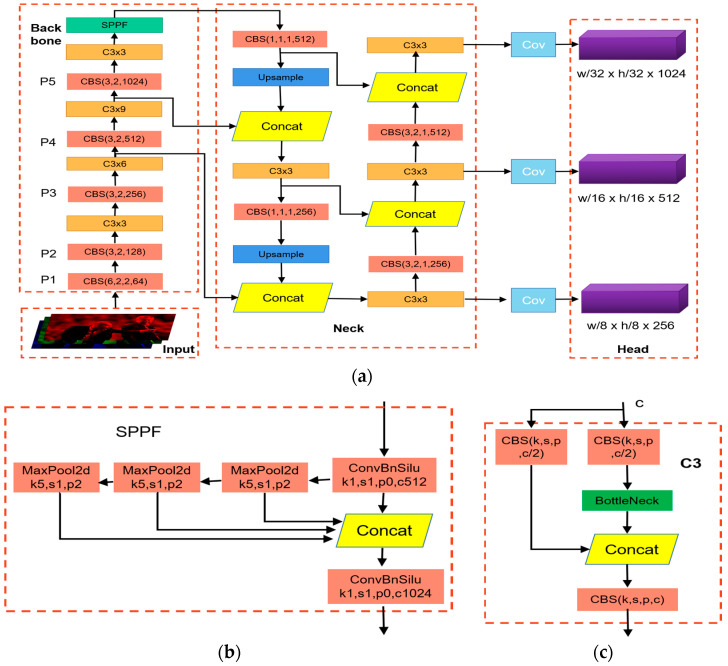
Network structure of YOLOv5. (**a**) Overall model framework with four components: Input, Backbone, Neck, and Head. Here, CBS is convolution, batch normalization, and SiLU activation, and C3 × *n* denotes *n* C3 layers. The image is initially fed into the Backbone structure for feature extraction. Subsequently, the Neck structure further integrates the extracted features, which are then input into the Head structure for final predictions. (**b**) Structural diagram of spatial pyramid pooling module. (**c**) C3 structure.

To clarify the feedforward process of the teacher and student detectors in Figure 2, we used YOLOv5 as a representative case. The feedforward process of object detection is shown in Figure 3. Assuming the input of the model is $F_{\mathrm{input}}^{B\times C\times H\times W}$, the input data are first passed through the teacher and student detectors to complete one forward propagation. Through that forward propagation, the neck output features $F_{T}^{1\times i}$ and $F_{S}^{1\times i}$ of the teacher and student detectors can be obtained, where i is the number of features fed to the detection head for final detection in the neck output features of the detector. Equations (1) and (2) represent the forward propagation process:

(1) $$F_{T}^{1\times i}=f_{\mathrm{te}}(F_{\mathrm{input}}^{B\times C\times H\times W},\theta_{\mathrm{te}})$$

(2) $$F_{S}^{1\times i}=f_{\mathrm{se}}\left(F_{\mathrm{input}}^{B\times C\times H\times W},\theta_{\mathrm{se}}\right),$$

where $f_{\mathrm{te}}$ and $f_{\mathrm{se}}$ are the feature encoding of the input data by the teacher and student detectors, respectively, and $\theta_{\mathrm{te}}$ and $\theta_{\mathrm{se}}$ are the model parameters corresponding to the relevant structure of the detector.

$F_{T}^{1\times i}$ and $F_{S}^{1\times i}$ are then fed into the proxy network. The final output of the proxy network is a feature vector of dimension 2 × k, represented as P = [$P_{T}^{j}$, $P_{S}^{j}$] (0 ≤ j ≤ k). Here, $P_{T}^{j}$ and $P_{S}^{j}$ are the probabilities of data passing through the teacher and student detectors, respectively, with values ranging from 0 to 1. $P_{T}^{j}$ = 0 indicates that data do not pass through the teacher detector, and the semantic features of that level are not distilled. Moreover, k is the number of semantic levels used for feature distillation. The decision process is described in Equation (3):

(3) $$P=\mathrm{Gumbel}\text{\_}\mathrm{Softmax}(f_{c}(F_{T}^{1\times i},F_{S}^{1\times i},\theta_{p})),$$

where the function $f_{c}$ is a fully connected operation and $\theta_{p}$ is the model parameter of the fully connected layer. “Gumbel softmax” is a method proposed in [42]. This method makes the sampling computation differentiable, allowing gradients to backpropagate to the proxy network during the backward propagation.

During the first forward process, $F_{S}^{1\times i}$ is also fed into the head of the student detector for detection. The detection loss $L_{\det}$ can be obtained using

(4) $$L_{\det}=f_{\det}(f_{h}(F_{S}^{1\times i},\theta_{h}),\mathrm{gt}),$$

where the function $f_{h}$ is the function of processing input features in the detection head, $\theta_{h}$ is the corresponding model parameter, gt is the ground truth, and $f_{\det}$ is the loss function of the student detector, which comprises three components: localization loss, classification loss, and object confidence loss.

Finally, the decision P obtained by Equation (3) is used to distill the semantic features of the *j*th level of the teacher detector for which $P_{T\;}^{j}$ ≠ 0. The distillation loss can be calculated as

(5) $$L_{\mathrm{KD}}^{j}=\mathrm{fgd}(F_{T}^{j},F_{s}^{j})\;(P_{T}^{j}\text{≠}0),$$

where fgd is the detector distillation method proposed by [21]. This method was used to calculate the feature distillation loss. Specifically, Equation (5) can be further expressed as

(6) $$L_{\mathrm{KD}}^{j}=\alpha L_{\mathrm{fg}}(F_{T}^{j},F_{s}^{j})+\beta L_{\mathrm{bg}}(F_{T}^{j},F_{s}^{j})+\gamma L_{\mathrm{at}}+\lambda L_{\mathrm{global}}(F_{T}^{j},F_{s}^{j})\;(P_{T}^{j}\text{≠}0),$$

where $L_{\mathrm{fg}}$ is the foreground distillation loss function for the feature maps, $L_{\mathrm{bg}}$ is the background distillation loss function, $L_{\mathrm{at}}$ is the attention loss function which enables the student detector to mimic the spatial and channel attention masks of the teacher detector, and $L_{\mathrm{global}}$ is the global distillation loss. The hyperparameters α, β, γ, and λ are used to balance the weights of each loss function.

Since $L_{\mathrm{KD}}^{j}$ is calculated from features with $P_{T}^{j}$ ≠ 0, the semantic level of the features used for distillation can be adaptively changed based on the decisions of the proxy network.

### 3.2. Routing Feedforward Process

Through the first forward progress, the detection loss $L_{\det}$ and the $L_{\mathrm{KD}}^{j}$ of the student detector are computed. The P output by the policy network was also obtained. However, the output of the proxy network depends on the output features of the neck structure of the student detector, which are constrained by the output of the policy network through $L_{\mathrm{KD}}^{j}$. Therefore, training both simultaneously is not suitable. The second round of forward propagation is used to address that problem.

At the preselected distillation feature level, the model determines the path through which the data flow based on P. When $P_{T\;}^{j}$ = 0, the data flow through the student detector, whereas when $P_{T}^{j}$ = 1, the data flow through the teacher detector. In contrast to [22], logits are not used for distillation, but the detection head of the teacher detector is used to output the final detection results of the routing network. The routing loss $L_{\mathrm{rout}}$ can be calculated as

(7) $$L_{\mathrm{rout}}=f_{\det\text{\_}t}(H_{\mathrm{rt}},\mathrm{gt}),$$

where $f_{\det\text{\_}t}$ is the detection loss function of the teacher detector, and $H_{\mathrm{rt}}$ is the output of the head of the teacher detector in the routing feedforward process.

### 3.3. Overall Loss

The overall loss function is

(8) $$L=L_{\det}+\sum_{j=1}^{k}L_{\mathrm{KD}}^{j}+L_{\mathrm{rout}}$$

Minimizing $L_{\det}$ and $L_{\mathrm{KD}}$ can make the student detector achieve higher detection performance, and minimizing $L_{\mathrm{rout}}$ can improve the proxy network decision making.

## 4. Experiments

### 4.1. Dataset

All experiments in this work were carried out on the Pascal visual object classes (VOC) dataset [43], which comprised 20 object categories. The training and validation sets of VOC2007 and VOC2012, totaling 16,551 images, were used as the training data for our experiments. For evaluation purposes, the test set of VOC2007, which included 4952 images, was used as the validation data. mAP_50_ and mAP_50–90_ were used as the evaluation metrics to assess the detection performance.

### 4.2. Experimental Details

The experiments in this work were carried out using the PyTorch 1.11.1 deep learning framework, with training performed on a device equipped with an Intel Xeon Platinum 8352 V CPU (Intel, Santa Clara, CA, USA) and 2 Nvidia A40 48 G GPUs (Nvidia, Santa Clara, CA, USA). The operating system was Ubuntu 20.04.

**Figure 4:**
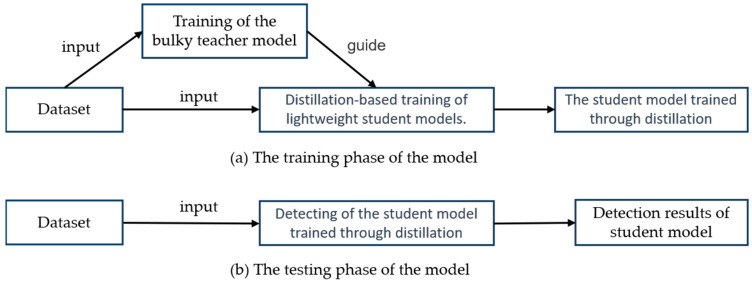
Distillation procedures. (**a**) Distillation training phase. During that phase, the teacher model was trained, and then the student model was trained, guided by the teacher model. (**b**) Testing phase. Here, the trained student model performed image detection and inference on the input image, with no involvement of the teacher model.

The distillation process is shown in Figure 4. The first step in distillation training was training a teacher model. This model was then used to direct the training procedure of the student model. In this study, YOLOv5 was used as the benchmark model. Three sets of experiments were carried out: benchmark experiments, distillation experiments of semantic features at different levels, and validation experiments of MSFAD. YOLOv5l, YOLOv5s, and YOLOv5n were first trained, with YOLOv5l serving as the teacher detector and YOLOv5s and YOLOv5n as the benchmark detectors. An exploratory experiment was then carried out to study the relation between the student detector’s performance and the distilled features’ semantic levels. Finally, the performance of the proposed MSFAD was verified. Table 1 provides detailed parameters for all the experiments, allowing readers to refer to and reproduce the experiments.

### 4.3. Comparison of Experimental Results

To evaluate the efficacy of the proposed method, a comparative analysis was carried out with widely adopted object detection distillation methods. Table 2 shows that the results demonstrate marked improvements achieved by the method. It yielded a 3.4% and 3.3% increase in mAP_50_ and a 3.3% and 2.2% enhancement in mAP_50–90_ for the student detector. Those advancements exceeded the performance gains observed in other prevalent distillation methods for student detectors. It was found that before distillation, the selected student detector’s performance was lower than that of the benchmark detector used for comparison. However, after using the MSFAD distillation, the detection accuracy of YOLOv5s surpassed that of all benchmark student detectors and most teacher detectors. Relative to the distillation results obtained using various benchmark methods, the MSFAD distillation approach produced a higher-performing YOLOv5s detector with substantially fewer parameters than the compared detectors. This outcome highlighted the substantial performance gains the MSFAD method brought to the student detector.

Table 3 compares the MSFAD-distilled model with other lightweight YOLO models. The results show that MSFAD-YOLOv5s outperformed YOLOv3-tiny by 23.3% in mAP_50_ and reduced the model parameters by 78.4%. Similarly, compared to YOLOv3-tiny, MSFAD-YOLOv5n achieved a 17.2% improvement in mAP_50_ with only 5.7% of the model parameters. It also improved the mAP_50_ by 4.7% while reducing the model parameters by 68.1%. MSFAD-YOLOv5n achieved the same detection accuracy as YOLOv4-tiny with only 8% of its parameters. Additionally, MSFAD-YOLOv5s outperformed YOLOv4-S by 1.7% in mAP_50_ while reducing the model parameters by 56.4%. Finally, MSFAD-YOLOv5s outperformed the latest YOLO model, YOLOv7-tiny, by 2.2% in mAP_50_ and 1.9% in mAP_50–90_, with a negligible increase in model parameters.

In summary, the proposed MSFAD method can substantially improve the detection accuracy of student detectors without adding model parameters.

### 4.4. Distillation of Semantic Features of Different Levels

To investigate how the semantic level of the distilled features affected the performance of the student detector, three exploratory experiments were carried out using the focal and global knowledge distillation (FGD) method proposed in [21]. YOLOv5s was selected as the student detector, and YOLOv5l as the teacher detector. The experimental conditions were as follows: (1) Distillation of features output only by the neck structure, (2) distillation of features output only by the backbone structure, and (3) distillation of the features output by both the backbone and the neck. Figure 3 shows the experimental results.

**Figure 5:**
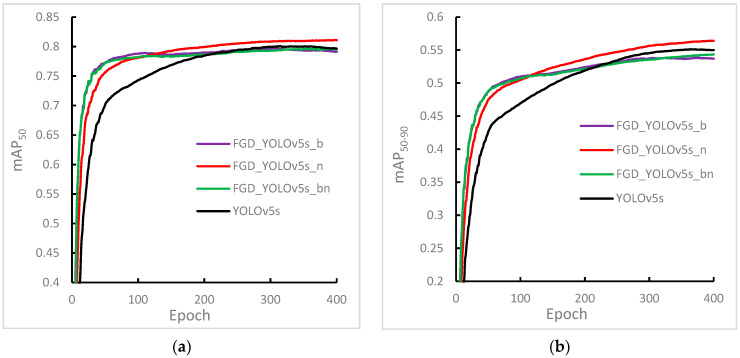
Experimental results of distilling features of various semantic levels of YOLOv5 using the focus group discussion method. (**a**) Experimental result of mAP_50_. (**b**) Experimental result of mAP_50–90_. Among them, YOLOv5s, FGD-YOLOv5s_b, FGD-YOLOv5s_n, and FGD-YOLOv5s_bn indicate that no distillation was carried out, and the features of the 4th, 6th, and 9th semantic level output by the backbone structure were selected for distillation. The features of the 17th, 20th, and 23rd semantic level output by the neck structure were distilled, and the features of the above six semantic levels were selected for distillation.

In comparing Figure 5a,b, consistent trends were found in the experimental results of mAP_50_ and mAP_50–90_, indicating that the experiments accurately identified the existing problems. Additionally, the comparison between FGD-YOLOv5s_n and YOLOv5s showed that the FGD distillation method was effective for the YOLO detector, with substantial improvements in the detection accuracy and convergence speed of the student detector.

Analyzing the results of FGD-YOLOv5s_b and FGD-YOLOv5s_n showed that selecting the output features of the backbone structure for distillation in the early training stage helped the student detector converge faster. This outcome indicates that the knowledge contained in the teacher detector’s shallow semantic features was more valuable in the initial training stage. As the knowledge accumulated, the value of the shallow semantic features gradually diminished, and the deep semantic features fused by the neck structure became more valuable for the student detector. Therefore, using deeper semantic-level features for distillation in the later training stage led to higher detection accuracy of the student detector. The findings suggest that the performance of the student detector was highly dependent on the semantic level of the distilled features.

Finally, comparing the results of FGD-YOLOv5s_b, FGD-YOLOv5s_bn, and YOLOv5s showed that selecting inappropriate semantic-level features for distillation at different training stages can have adverse effects. Specifically, both FGD-YOLOv5s_b and FGD-YOLOv5s_bn achieved less effective results than those of YOLOv5s without distillation in the later stages of distillation training.

### 4.5. Visual Analysis of Feature Maps

**Figure 6:**
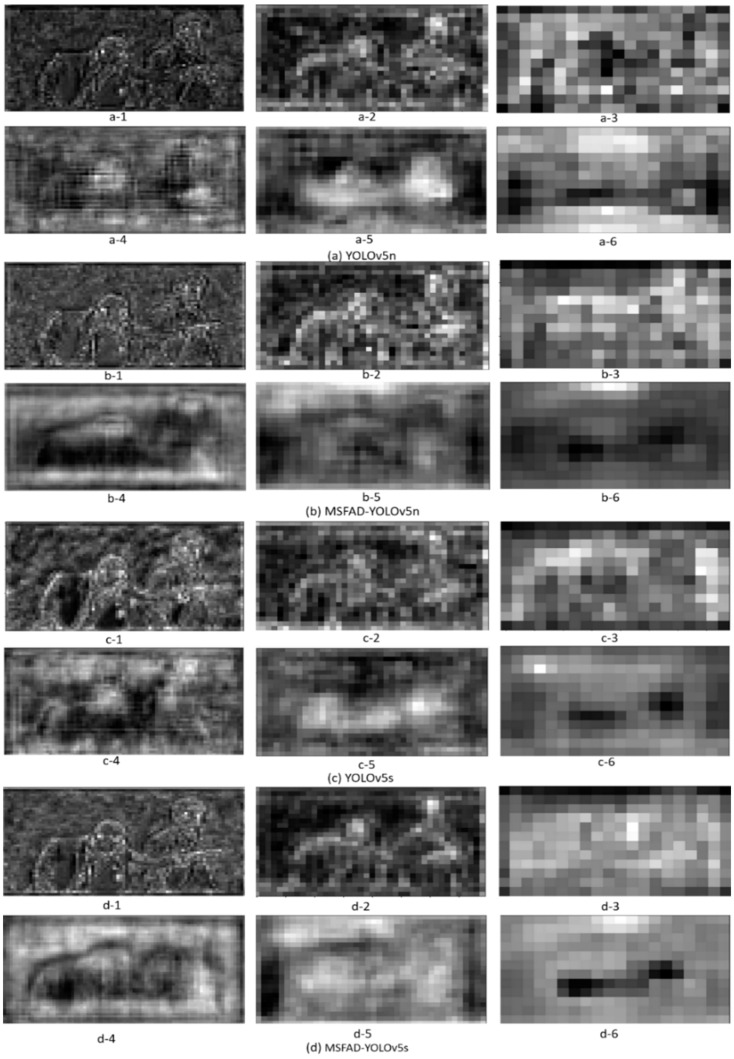
Features of student detector output before and after distillation. In each group of six maps, the first three were output by the backbone and the second three by the model head. (**a**,**c**) Feature maps of the detector’s output before distillation. (**b**,**d**) Feature maps after MSFAD distillation.

To determine the effectiveness of the MSFAD method, the feature maps of each model were visualized before and after distillation for the feature maps output by the backbone structure and neck structure. Figure 6 shows the visualization results, where in each group of six images, the first three feature maps were output by the backbone and the second three by the model head. The results indicate that the distillation process markedly improved the feature maps of the model. Specifically, the model head feature maps b-1, b-2, d-1, and d-2 after distillation had more precise features and a better suppression of background noise than the backbone feature maps a-1, a-2, c-1, and c-2 before distillation. Furthermore, b-3 and d-3 after distillation extracted more abstract semantic features than a-3 and c-3, demonstrating that the backbone had more robust feature extraction capability after distillation. Similarly, compared to the feature maps a-4, a-5, c-4, and c-5 output by the head before distillation, the feature maps b-4, b-5, d-4, and d-5 after distillation had cleaner backgrounds, enabling a clearer representation of the posture of the two people. Moreover, b-6 and d-6 after distillation displayed the position information of the two people more clearly and accurately than did a-6 and c-6, with better foreground and background separation.

Visualizing feature maps can gain deeper insights into how the distilled student detector achieved high performance. Compared to feature maps output by the student detector before distillation, the proposed MSFAD method effectively reduced background noise interference. Additionally, the MSFAD method enhanced the student detector’s feature extraction ability.

**Figure 7:**
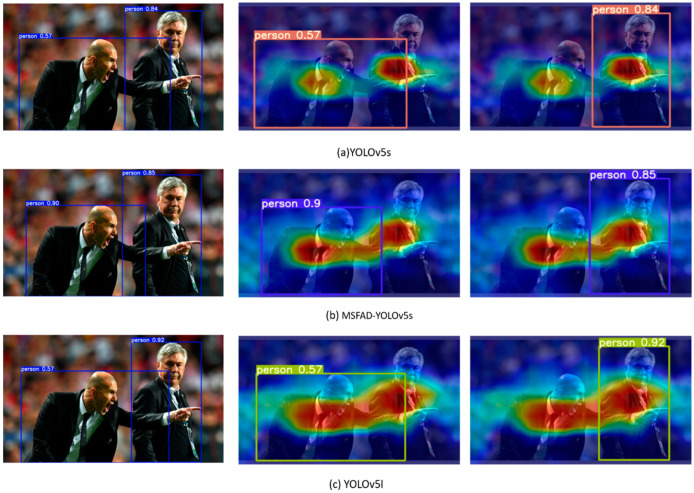
Detection results and corresponding feature heat maps of each model. In each image pair, the left side shows the inference result of the model, and the middle and right side shows the feature area that the inference result focused on for the detection category. The color intensities of each region indicate the contribution of those regions to the detection category, with darker regions indicating greater contributions.

To verify the actual performance of the student detector distilled using the MSFAD method, the detection performances of the models were compared before and after distillation. Heat maps of the detected regions visualized with the use of gradient-weighted class activation mapping (Grad-CAM) [44] are shown in Figure 7. The images in the left column demonstrate the inference results of each model, showing that MSFAD-YOLOv5s distilled by the MSFAD method showed substantially higher detection accuracy than that of the detector before distillation, with the detection accuracy of the person on the left in the images even exceeding that of the teacher detector. The images in the right column display the target regions to which the detector paid attention, demonstrating that the regions of interest of MSFAD-YOLOv5s after distillation were highly similar to those of the teacher network. Relative to the detector before distillation, the target regions to which MSFAD-YOLOv5s paid attention are more precise in position and darker in color. Those results indicate that the MSFAD method can help the student detector accurately identify the features of target categories, leading to higher detection accuracy.

### 4.6. Ablation Study

#### 4.6.1. Study of Different Distillation Points

In this section, we name various distillation points to demonstrate the effectiveness of our approach. Specifically, we used a state-of-the-art FGD distillation method for comparison. The experimental results are summarized in Table 4.

The results in Table 4 show that regardless of the feature selected for distillation, the proposed MSFAD method consistently outperformed the FGD method in enhancing the performance of student detectors.

Comparing the results of FGD-YOLOv5s-b, MSFAD-YOLOv5s-b, FGD-YOLOv5n-b, and MSFAD-YOLOv5n-b to YOLOv5s and YOLOv5n, we observed that using the FGD method to distill Backbone output features resulted in a degradation in the performance of the student detector compared with the predistillation stage. This indicates that the FGD method was ineffective in mitigating the negative effect of Backbone output features on the student detector during the later stages of model training. In contrast, the MSFAD method timely mitigated that negative effect based on varying training stages and samples, ensuring that the post-distillation performance remained stable.

Finally, comparing the results of FGD-YOLOv5n-n, MSFAD-YOLOv5n-n, FGD-YOLOv5s-n, and MSFAD-YOLOv5s-n showed that Neck output features provided more valuable guidance for student detectors compared with Backbone features. The MSFAD technique effectively harnessed the latent knowledge within the teacher detectors to guide the learning process of student detectors, thereby facilitating higher performance.

#### 4.6.2. Stability Analysis of Student Models before and after Distillation

This section describes the investigation of the student detector’s stability before and after distillation.

**Figure 8:**
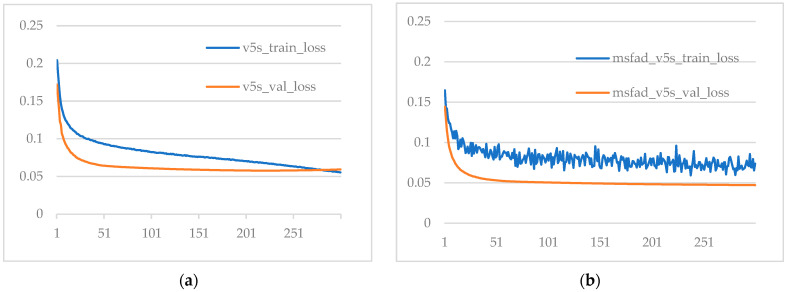
Comparison of the loss between the training and validation sets of the proposed model before and after distillation. (**a**) Loss of the model before distillation. (**b**) Loss of the model after distillation.

Figure 8 shows the loss curves of the model before and after distillation on the training and validation sets. Comparing Figure 8a,b, it can be concluded that the student detector after MSFAD distillation did not have overfitting. On the contrary, the loss of the student detector after distillation on the validation set was less than that of the model before distillation, indicating that the model after distillation performed better on the validation set.

**Figure 9:**
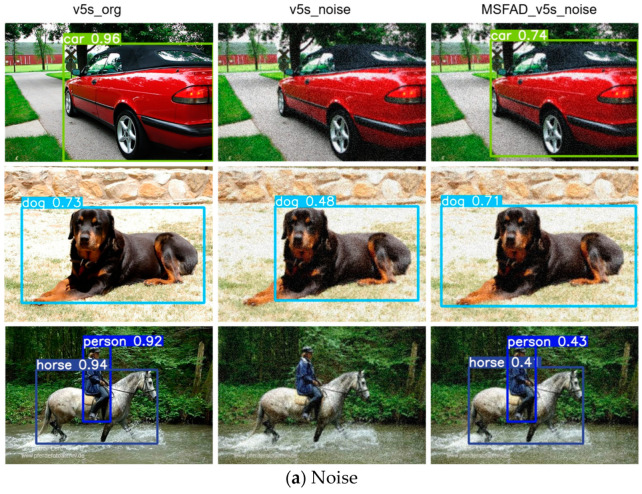

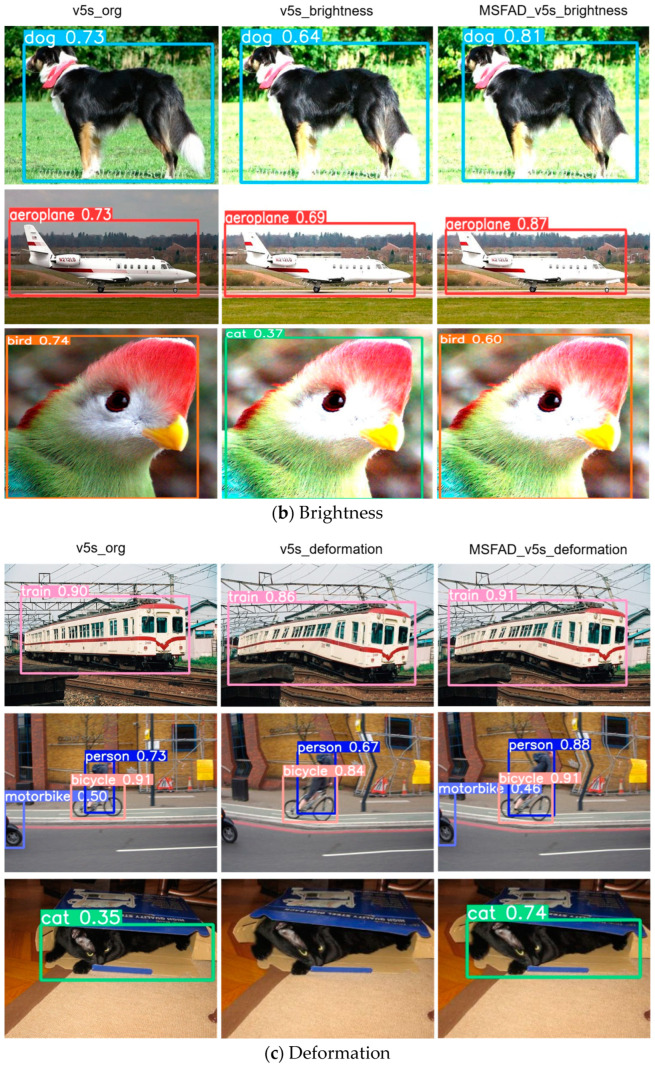
Stability analysis results for the proposed model before and after distillation. Left columns show results of detecting normal images using models without distillation. Middle columns show results of using non-distilled models to detect images with disturbances. Right columns show results of detecting perturbed images using the MSFAD-distilled model. (**a**) Model’s detection performance under the influence of added noise. (**b**) Model’s response to changes in image brightness. (**c**) Model’s behavior when confronted with image deformation.

Figure 9 shows a stability analysis of the student detector before and after distillation. We examined its performance under three perturbation methods: introducing noise to the image, altering image brightness, and deforming the image. To highlight the improved stability resulting from distillation, we initially used the non-distilled model to detect normal images as a baseline for comparison. Then, we used the non-distilled model to detect noisy images to assess its resilience to interference. Finally, we applied the MSFAD-distilled model to detect perturbed images, confirming the enhanced stability achieved through the MSFAD method. We selected images with diverse backgrounds and targets to ensure a comprehensive evaluation of our experiments.

Figure 9a shows the marked decline in detection performance of the non-distilled model when exposed to noise disturbances, to the extent that it failed to detect targets within the image. In contrast, the model distilled with MSFAD effectively mitigated the effect of noise, resulting in a marked improvement in model stability against noise interference.

Figure 9b highlights the discernible reduction in detection accuracy of the nondistilled model when the image’s brightness was adjusted. This decline became evident when a bird was inaccurately identified as a cat. Conversely, the distilled model adeptly mitigated the unfavorable effects of brightness modifications. Remarkably, the detection accuracy of specific targets exceeded that achieved during normal image detection. This pronounced improvement shows the increased stability of student detectors when confronted with fluctuations in brightness.

Figure 9c clearly shows that image deformation markedly undermined the detection accuracy of the non-distilled model, occasionally causing missed detections. In contrast, the MSFAD-distilled model effectively handled image deformation, resulting in substantially enhanced detection accuracy relative to that of the non-distilled model in normal image detection. This observation underscored the distilled model’s resilience to image deformation.

In conclusion, the student detector, derived from the MSFAD distillation procedure, had substantial resilience against interference. This capability improved the model’s ability to handle complex environmental scenarios.

## 5. Conclusions

This study demonstrated the negative effect on the student detector’s performance of selecting inappropriate semantic-level features for distillation during various training stages. To address this problem, the MSFAD method is proposed, which includes a routing network for the teacher and student detectors and a proxy network for decision making. The method enables the student detector to automatically select appropriate semantic levels to learn from based on the current training stage and training samples. The effectiveness of the proposed method was validated on the YOLOv5 model. The experimental results found substantial performance gains for the student detector over state-of-the-art FGD. The proposed method increased the mAP_50_ of YOLOv5s by 3.4% and the mAP_50–90_ by 3.3%. Moreover, YOLOv5n, with only 1.9 M parameters, achieved detection performance comparable to that of YOLOv5s. Compared to feature maps output by the student detector before distillation, the proposed MSFAD method reduced background noise interference and enhanced the student detector’s feature extraction ability.

Our method outperformed mainstream object detection distillation algorithms and delivered substantial performance enhancements to student detectors. However, training the model through distillation demanded substantial graphics memory allocation. For instance, when configuring the batch size to eight and the input image dimensions to 640 × 640, a considerable 38 G of graphics memory became indispensable, and the entire training process consumed approximately 1 week. Future work will address the problem of high memory requirements in feature-based distillation methods during the distillation training process. This is a critical problem that limits the advancement of KD techniques.

## Figures and Tables

**Table 1:** Details of experiments.

Name	Epoch	lr	Weight_Decay	Momentum	Batch	Img_Size	T	α	β	γ	λ
YOLOv5l	300	0.01	0.0005	0.937	16	640	–	–	–	–	–
YOLOv5s	400	0.01	0.0005	0.937	16	640	–	–	–	–	–
YOLOv5n	400	0.01	0.0005	0.937	16	640	–	–	–	–	–
FDG ^a^-YOLOv5s_b	400	0.01	0.0005	0.937	16	640	0.5	1 × 10^−3^	5 × 10^−4^	5 × 10^−4^	5 × 10^−6^
FGD ^a^-YOLOv5s_n	400	0.01	0.0005	0.937	16	640	0.5	1 × 10^−3^	5 × 10^−4^	5 × 10^−4^	5 × 10^−6^
FGD ^a^-YOLOv5s_bn	400	0.01	0.0005	0.937	16	640	0.5	1 × 10^−3^	5 × 10^−4^	5 × 10^−4^	5 × 10^−6^
FGD ^a^-YOLOv5n	400	0.01	0.0005	0.937	16	640	0.5	1 × 10^−3^	5 × 10^−4^	5 × 10^−4^	5 × 10^−6^
MSFAD-YOLOv5s	400	0.01	0.0005	0.937	16	640	0.5	1 × 10^−3^	5 × 10^−4^	5 × 10^−4^	5 × 10^−6^
MSFAD-YOLOv5n	400	0.01	0.0005	0.937	16	640	0.5	1 × 10^−3^	5 × 10^−4^	5 × 10^−4^	5 × 10^−6^

^a^ Focal and global knowledge distillation proposed in [21].

**Table 2:** Comparison of various distillation methods on the visual object classes dataset. The results of the benchmark methods used for comparison are from [38].

Method	Params (M)	mAP_50_ (%)		mAP_50–90_ (%)	
Faster R-CNN-Res101 (teacher)	232	82.8	Improvement	56.3	Improvement
Faster R-CNN-Res50 (student)	159	82.2		54.2	
+Mimicking [18]	159	82.3	+0.1	55.5	+1.3
+Fine-grained [19]	159	82.2	=	55.4	+1.2
+Fitnet [11]	159	82.2	=	55.1	+0.9
+GID [38]	159	82.6	+0.4	56.5	+2.3
RetinaNet-Res101 (teacher)	217	81.9	Improvement	57.3	Improvement
RetinaNet-Res50 (student)	72.7	80.9		55.4	
+Fine-grained [19]	72.7	81.5	+0.6	56.6	+1.2
+Fitnet [11]	72.7	81.4	+0.5	55.8	+0.4
+GID [38]	72.7	82.0	+1.1	57.9	+1.3
FCOS-Res101 (teacher)	196	81.6	Improvement	58.4	Improvement
FCOS-Res50 (student)	123	80.2		56.1	
+Fitnet [11]	123	80.3	+0.1	57.0	+0.9
+GID [38]	123	81.3	+1.1	58.4	+2.3
YOLOv5l (teacher)	46.5	84.6	Improvement	63.1	Improvement
YOLOv5s (student)	7.2	79.1		54.0	
+MSFAD (ours)	7.2	82.5	+3.4	57.3	+3.3
YOLOv5l (teacher)	46.5	84.6	Improvement	63.1	Improvement
YOLOv5n (student)	1.9	73.1		46.6	
+MSFAD (ours)	1.9	76.4	+3.3	48.8	+2.2

**Table 3:** Performance comparison of YOLO lightweight detection algorithm.

Model	P (%)	R (%)	mAP_50_ (%)	mAP_50–90_ (%)	Params (M)
YOLOv3-tiny	61.5	55.1	59.2	–	17.5
YOLOv4-tiny	79.3	76.0	77.8	–	22.6
YOLOv4-S	78.9	80.1	80.8	–	16.5
YOLOv7-tiny	79.2	76.7	80.3	55.4	6.2
MSFAD-YOLOv5s	80.5	79.8	82.5	57.3	7.2
MSFAD-YOLOv5n	74.5	73.1	76.4	48.8	1.9

**Table 4:** Experimental results of using global knowledge distillation (FGD) and multilayer semantic feature adaptive distillation (MSFAD) methods to distill the features of different distillation points. FGD-YOLOv5s-n and MSFAD-YOLOv5s-n denote the distillation of Neck output feature maps of YOLOv5s using FGD and MSFAD, respectively. FGD-YOLOv5s-b and MSFAD-YOLOv5s-b denote the distillation of Backbone output feature maps of YOLOv5s using FGD and MSFAD, respectively. FGD-YOLOv5n-b and MSFAD-YOLOv5n-b denote the distillation of Backbone output feature maps of YOLOv5n using FGD and MSFAD, respectively.

Model	P (%)	R (%)	mAP_50_ (%)	mAP_50–90_ (%)	Params (M)
YOLOv5l (teacher)	84.6	78.8	84.6	63.1	46.5
YOLOv5s (student)	80.4	73.1	79.1	54.0	7.2
YOLOv5n (student)	73.2	69.6	73.1	46.6	1.9
FGD-YOLOv5n-n	74.1	72.8	75.0	47.6	1.9
MSFAD-YOLOv5n-n	74.5	73.1	76.4	48.8	1.9
Improvement	+0.4	+0.3	+1.4	+1.2	–
FGD-YOLOv5s-n	79.5	78.9	81.1	56.6	7.2
MSFAD-YOLOv5s-n	80.5	79.8	82.5	57.3	7.2
Improvement	+1.0	+0.9	+1.4	+0.7	–
FGD-YOLOv5s-b	78.8	76.1	78.2	53.1	7.2
MSFAD-YOLOv5s-b	79.7	75.9	79.0	54.1	7.2
Improvement	+0.9	−0.2	+0.8	+1.0	–
FGD-YOLOv5n-b	72.8	68.7	72.5	45.8	1.9
MSFAD-YOLOv5n-b	73.7	70.2	73.3	46.5	1.9
Improvement	+0.9	+1.6	+0.8	+0.7	–

## Data Availability

Two publicly available datasets (Pascal VOC 2007, Pascal VOC 2012) were used to illustrate and evaluate the proposed method. Our code is available at https://github.com/ljq6688/msfad/tree/master (accessed on 24 August 2023).
